# Comparative growth of spotted fever group *Rickettsia* spp. strains in Vero cells

**DOI:** 10.1590/0074-02760160093

**Published:** 2016-08

**Authors:** Arannadia Barbosa Silva, Myrian Morato Duarte, Vinicius Figueiredo Vizzoni, Ana Íris de Lima Duré, Diego Montenegro Lopéz, Rita de Maria Seabra Nogueira, Carlos Augusto Gomes Soares, Erik Machado-Ferreira, Gilberto Salles Gazêta

**Affiliations:** 1 Fundação Oswaldo Cruz, Instituto Oswaldo Cruz, Programa de Pós-Graduação em Biodiversidade e Saúde, Rio de Janeiro, RJ, Brasil; 2 Fundação Oswaldo Cruz, Instituto Oswaldo Cruz, Laboratório de Referência Nacional em Vetores das Riquetsioses, Rio de Janeiro, RJ, Brasil; 3 Fundação Ezequiel Dias, Serviço de Virologia e Riquetsioses, Belo Horizonte, MG, Brasil; 4 Universidade Federal do Rio de Janeiro, Instituto de Biologia, Laboratório de Genética Molecular de Eucariontes e Simbiontes, Rio de Janeiro, RJ, Brasil; 5 Fundação Oswaldo Cruz, Instituto Oswaldo Cruz, Laboratório de Doenças Parasitárias, Rio de Janeiro, RJ, Brasil; 6 Universidade Estadual do Maranhão, Curso de Medicina Veterinária, Laboratório de Parasitologia, São Luís, MA, Brasil

**Keywords:** tick-borne disease, rickettsial biology, growth kinetics

## Abstract

In Brazil, the spotted fever group (SFG) *Rickettsia rickettsii* and *Rickettsia parkeri* related species are the etiological agents of spotted fever rickettsiosis. However, the SFG, *Rickettsia rhipicephali*, that infects humans, has never been reported. The study of growth dynamics can be useful for understanding the infective and invasive capacity of these pathogens. Here, the growth rates of the Brazilian isolates *R. rickettsii* str. Taiaçu, *R. parkeri* str. At#24, and *R. rhipicephali* HJ#5, were evaluated in Vero cells by quantitative polymerase chain reaction. *R. rhipicephali* showed different kinetic growth compared to *R. rickettsii* and *R. parkeri.*

In Brazil, the spotted fever group (SFG) *Rickettsia rickettsii* and *Rickettsia parkeri* related species are the etiological agents of spotted fever rickettsiosis. *R. rickettsii* is the causative agent of Rocky Mountain spotted fever (RMSF) and Brazilian spotted fever (BSF), which is considered the most severe of all tick-borne rickettsiosis (Parola et al. 2005). *R. parkeri* was recently reclassified as a pathogenic bacterium that causes an eschar-associated rash illness, considered less severe than BSF (Paddock et al. 2004). *Rickettsia rhipicephali* of the SFG that infects humans has never been reported; however, in vitro experiments have shown this bacterium to be moderately pathogenic in guinea pigs (Burgdorfer et al. 1978, Gage & Jerrells 1992).

Until now, very few studies have characterised the growth dynamics of different species or strains of *Rickettsia* in culture media and provided parameters to advance the knowledge on this pathogen (Eremeeva et al. 2003, Boldis et al. 2009). In this context, comparative analyses of *R. rhipicephali* and pathogenic SFG rickettsiae could be useful to provide new information about the pathogenic potential of this species. Thus, in the present study, we evaluated and compared the growth rate of the Brazilian isolates *R. rickettsii* str. Taiaçu (Pinter & Labruna 2006), *R. parkeri* str. At#24 (Silveira et al. 2007), and *R. rhipicephali* str. HJ#5 (Labruna et al. 2007) after infection of Vero cells.

Experiments were performed in the biosafety level 3 laboratory of Divisão de Epidemiologia e Controle de Doenças (DECD) of Fundação Ezequiel Dias - FUNED, Belo Horizonte, Minas Gerais, Brazil. The *Rickettsia ompA* and *gltA* genes were amplified using the primer sets Rr190.70p/Rr190.602 and CS-78/CS323 (Regnery et al. 1991, Labruna et al. 2004) and sequenced to confirm the identity of these *Rickettsia* strains (Data not shown). In brief, cryogenic tubes containing *Rickettsia*-infected Vero cells were rapidly thawed, and their contents were added to flasks with an uninfected Vero cell monolayer, and incubated at 28ºC without CO_2_. After two passages, the confluent monolayer was scraped, and the infection rate was measured by quantitative polymerase chain reaction (qPCR) normalising to initial inoculums. At this point, Vero cells with bacteria were partially 10 purified with syringes (Ammerman et al. 2008) and added to bottles containing an equal 11 amount of uninfected Vero cells in a confluent monolayer. The flasks were incubated at 28ºC without CO_2_ for 1, 2, 24, 48 and 72 h. Cell infection was monitored by Giménez (1964) staining at 24, 48 and 72 h. Genomic DNA extraction from 100 µL of cells in suspension was performed using an Illustra RNAspin Mini RNA Isolation kit (GE Healthcare®) without RNase addition, according to the standard operating procedure (FUNED). Additional DNA samples from Vero cells infected with *R. parkeri*, *R. rhipicephali* and *Rickettsia amblyommii* were obtained using the extraction method described previously, using a QIAamp DNA Blood Mini Kit (Qiagen®) and a High Pure Viral Nucleic Acid Kit (Roche Applied Science®), to test quality by qPCR. DNA samples were quantified using a NanoVue Plus spectrophotometer (GE Healthcare Bio-Sciences AB®), and DNA integrity was analysed by 1% agarose gel electrophoresis (Data not shown).

qPCR reactions were performed using DNA samples from *Rickettsia*-infected Vero cells and a SYBR® Green PCR Master Mix (Applied Biosystems®) as recommended by the manufacturer. Each qPCR assay contained 30 ng of template DNA and primers for *ompA* (RR190.588F/RR190.701R) and reference (ACTB-F/ACTB-R) genes at a final concentration of 0.4 mM (Eremeeva et al. 2003, Ahn et al. 2008). PCR conditions were as follows: 95ºC for 10 min (hot-start), 40 cycles (95ºC for 15 s and 60ºC for 1 min). Amplification, data acquisition and data analysis were performed with a 7500 fast real-time PCR System (Applied Biosystems®). Comparative analysis of the *Rickettsia* spp. load in Vero cells was performed using C_T_ values for each sample (culture of Vero cells and *Rickettsia*, 1, 2, 24, 48 and 72 h post-inoculation), using the equation for 2^-∆∆CT^, in which ∆∆C_T_ = (C_T *ompA*_ - C_T*ßactin*_) _timex_ - (C_T*ompA*_ - C_T*ßactin*_) _time0_ (Livak & Schmittgen 2001). For the applied ∆∆C_T_ calculation, primer efficiencies were determined using a standard curve developed from template DNA at concentrations of 5, 10, 30, 50 and 100 ng/µL (Supplementary Figure).

The experiments presented here were conducted using two or three biological replicates, which were analysed in triplicate. We used the percentage of infected cells as the dependent variable, and time and *Rickettsia* species as independent variables. Central tendency measures and distribution were calculated and significant differences were assessed (ANOVA) for multiple comparisons; Fisher’s least significant difference (LSD) tests between treatments were developed with Statgraphics Centurion XVI (Statpoint Technologies 2006). For all significant differences, the 95% confidence interval (CI) and homoscedasticity of the variance were tested (Levene’s test).

The growth rate of these bacterial strains was initially analysed by optical microscopy. *Rickettsia*-like structures were observed in Giménez-stained Vero cells (Fig. 1A). Considering viable Vero cells (based on nucleus integrity), the number of infected cells (or those with attached *Rickettsia*) was counted after 24, 48 and 72 h after bacterial inoculation (Fig. 1). Interestingly, Vero cell infectivity was higher for *R. rhipicephali* than for the other two species at 24 (higher difference), 48 and 72 h post-inoculation. Moreover, at 72 h post-inoculation, the highest percentage of infected cells, 98.92%, 91.48% and 99.82%, was observed for *R. rickettsii*, *R. parkeri* and *R. rhipicephali*, respectively (Fig. 1B).

**Fig. 1:**
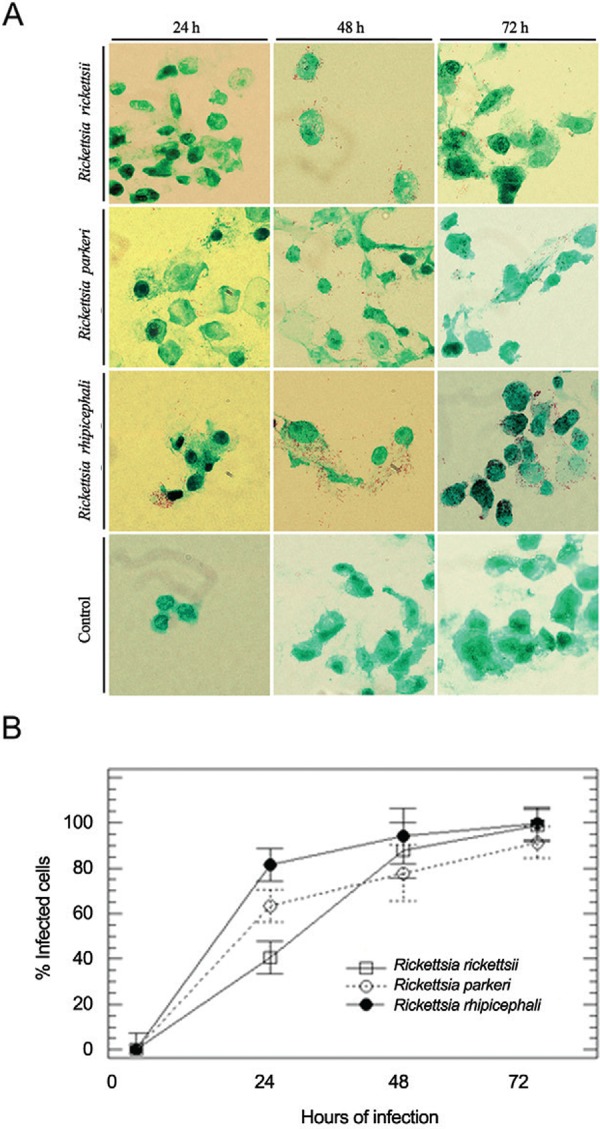
: visualization of Vero cells infected with spotted fever group *Rickettsia* spp. strains. (A) Photomicrographs illustrating the presence of *Rickettsia* spp. in Vero cells and uninfected Vero cells (Control) stained according to the Giménez method (Giménez 1964) (1000× magnification, optical microscope Olympus DP72) at 24, 48 and 72 h post bacterial inoculation; (B) percentage of Vero cells infected with *R. rickettsii* str. Taiaçu, *R. parkeri* str. AT#24, and *R. rhipicephali* str. H#J5 at the same time points. The results were statistically significant (Time course F3,18 = 223,56; p = 0.000; Species F2,18 = 5,10; p = 0.018; interaction, F6,18 = 4,43; p = 0,006; 95% CI; Levene’s = 0.07).

Four primer pairs for the rickettsial *ompA* gene and three primer pairs for eukaryotic genes of *ß-actin* and ribosomal protein L13A and L32 were tested by qPCR amplification (Eremeeva et al. 2003, Ahn et al. 2008). The best primer pairs for *Rickettsia* (RR190.588F/RR190.701R) and eukaryotic cells (ACTB-F/ACTB-R) were obtained through melt curve analysis (data not shown). For the comparative C_T_ method to be valid, the amplification efficiencies of the target rickettsial *ompA* gene and the reference eukaryotic *ß-actin* gene must be approximately equal (Livak & Schmittgen 2001). To validate this method, we prepared a dilution series of DNA template obtained from uninfected and *Rickettsia*-infected Vero cells. The reaction efficiencies for each DNA sample/primer set were evaluated based on slopes of the regression lines for C_T_ versus the relative dilution series (Supplementary Figure). The slopes of the regression lines for ∆C_T_ versus DNA template dilution were within the range of -0.1 to +0.1, confirming the validity of the relative quantification method (Supplementary Table).

The relative amount of *Rickettsia* in eukaryotic cells was determined by *ompA*/*ß-actin* qPCR analysis over a 72 h time course of infection in Vero cells. It was evident that the amount of Vero cell-infecting *Rickettsia* increased with time, reaching the highest loads at 72 h post-inoculation (Fig. 2). Utilising the computed 2^-∆∆CT^ values, *R. rhipicephali* numbers increased by approximately 8-, 4-, 3.8- and 17-fold during the 72 h time course, as shown in Fig. 2A. Based on comparative analysis, *R. rhipicephali* presented a distinct behaviour, with infectivity approximately 4.7-, 8.5-, 3.1- and 2.8-fold greater than that of pathogenic *R. rickettsii* at 2, 24, 48 and 72 h post-inoculation, respectively (Fig. 2B). Significant differences (F_2,65_ = 492,37; p = 0.000; 95% CI) were identified based on the bacteria/Vero cell proportion when the three species used in this study were compared; these differences were more evident at 72 h of infection (Fig. 2). DNA samples utilised in these analyses were predominantly purified using the RNA isolation kit (GE Healthcare). Comparative C_T_ analysis utilising two additional DNA isolation kits demonstrated no statistically significant difference (F_2,51_ = 0,51, p = 0.603, 95% CI, Levene’s p = 0.451) between the yield and quality of DNA obtained by these kits. Taken together, these data suggest that *R. rhipicephali* exhibited faster growth in cell culture over 72 h, when compared to *R. rickettsii* and *R. parkeri* strains.

The invasion process of SFG *Rickettsia conorii* in Vero cells occurs only a few minutes after *Rickettsia*-host cell adhesion, and proceeds via induced phagocytosis and subsequent intracytoplasmic release through the lysis of phagosomal membranes (Teysseire et al. 1995). In this work, the processes of *Rickettsia*-host cell contact and entry into Vero cells were not assessed; however, the quantitative (relative) data demonstrated that after 2 h, the number of *Rickettsia*-infected Vero cells was 1.3-, 1.6- and 8-fold higher than that 1 h post-inoculation with *R. rickettsii*, *R. parkeri* and *R. rhipicephali*, respectively. Thus, it suggested that the processes of adhesion, entry and escape to the cytoplasm were faster with *R. rhipicephali* inoculation, which would provide additional time for bacterial cell division (Figs 1B, 2A). Interestingly, *Rickettsia rickettsii* str. Sheila Smith was shown to reach its highest level of multiplication at 72 h post-inoculation in Vero cells (Noriea et al. 2015). Meanwhile, *Rickettsia slovaca* reached its highest level after 96 h post-inoculation (Boldis et al. 2009). To better evaluate the kinetic growth of *R. rhipicephali* compared to that of *R. rickettsii* and *R. parkeri*, additional studies using a time course of 14 days, which covers exponential, stationary and decline growth phases, should be performed.

To be pathogenic in mammals, tick-borne bacteria must be able to survive in the tick vector, be transmitted during tick feeding, avoid or subvert the host immune responses, replicate in host organisms; and spread to new hosts. In this scenario, *R. rhipicephali* has some of these characteristics; this species has been frequently described to infect ticks of different genera including *Rhipicephalus* spp., *Dermacentor* spp., and *Haemaphysalis juxtakochi* (Philip et al. 1981, Labruna et al. 2007, Hsu et al. 2011). Moreover, direct inoculation of *R. rhipicephali* into guinea pigs and voles resulted in a less severe rickettsiosis than that caused by *R. rickettsii* (Burgdorfer et al. 1978, Gage & Jerrells 1992), indicating that *R. rhipicephali* are able to survive inside the host organism, using mechanisms to evade or overcome the host immune system. Nonetheless, to consider *R. rhipicephali* as a new SFG pathogen, additional studies including those using different tick vector species, different vertebrate hosts and more sensitive molecular tools are needed. In contrast, Norment and Burgdorfer (1984) detected no clinical signs in dogs that were exposed to ticks infected with *R. rhipicephali.* It should be noted that *R. rhipicephali* str. HJ#5 was isolated from Vero cell culture in 2005 (Labruna et al. 2007). Thus, the differential growth of *R. rhipicephali* in Vero cells could be more related to its ability to adapt to this host cell line than its pathogenic potential, as was previously observed for *Rickettsia prowazekii* infection of chicken embryo cells (Wisseman & Waddell 1975).

Some members of the SFG have never been associated with human and animal diseases (Parola et al. 2013); however, it should be noted that some current human pathogenic species were first classified as non-pathogenic or of unknown pathogenicity. This fact deserves attention because it denotes the possibility of human infection in the future. Thus, studies on the growth dynamics of *Rickettsia* sp. are useful for understanding the infective and invasive capacity of these pathogens.

**Fig. 2:**
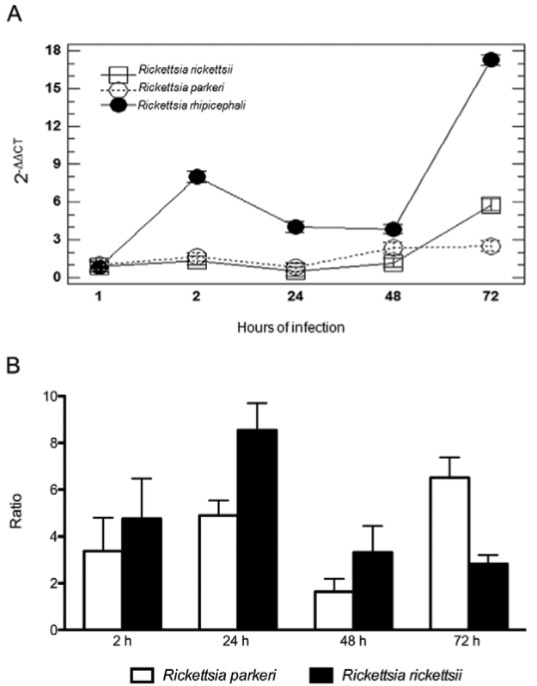
: relative quantification of spotted fever group *Rickettsia* spp. strains in Vero cells. (A) Relative growth curves of *R. rickettsii* str. Taiaçu, *R. parkeri* str. AT#24, and *R. rhipicephali* str. H#J5 in Vero cells at 1, 2, 24, 48 and 72 h post bacterial inoculation. There was a statistically significant difference between species [F4,65 = 304,90; p = 0.000; 95% confidence interval (CI)], an interaction between time and species (F8,65 = 114,50; p = 0.000; 95% CI). (B) Comparative ratio of *R. rhipicephali* abundance to those of *R. rickettsii* and *R. parkeri,* after infection. Comparisons with *R. parkeri* (white bars) and *R. rickettsii* (black bars) are presented. *Rickettsia* sp. abundances were determined as the relative amount of bacterial *ompA* / eukaryotic *ß-actin* at 2, 24, 48 and 72 h.
