# Supplementary material for: Comparative growth of spotted fever group Rickettsia spp. strains in Vero cells
Source: Mem Inst Oswaldo Cruz. 2016 Aug;111(8):528–31. doi: 10.1590/0074-02760160093 (PMC4981112; doi:10.1590/0074-02760160093)
Supplement: Supplementary file 1 [file 0074-0276-mioc-0074-02760160093-sd.pdf]

Mem Inst Oswaldo Cruz, Rio de Janeiro, Vol. *111*(8): 1-1, August 2016

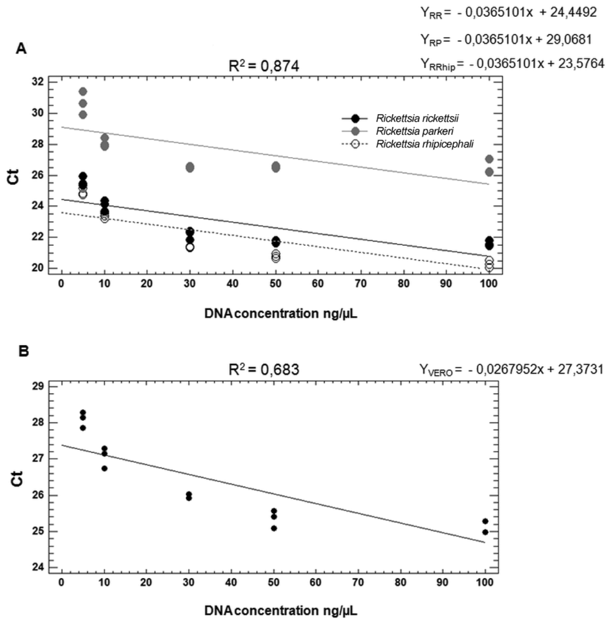

Standard curves based on quantitative polymerase chain reaction (qPCR)analyses of a five-fold (5, 10, 30, 50 and 100 ng/µL) DNA dilution series using primers for Rickettsial *ompA* gene and eukaryotic *ß-actin*. (A) Results from the Rickettsial *ompA* primer set (190.588F-190.701R) and DNA samples from *R. rickettsii*, *R. parkeri* and *R. rhipicephali*; (B) results from the eukaryotic *ß-actin* primer set and Vero cell DNA samples. Each qPCR assay contained 30 ng/µL of template DNA; primers were used at a final concentration of 0.4 mM. Ct: cycle threshold; RR: *R. Rickettsii* str. Taiaçu; RP: *R. parkeri* str. AT#24; RRhip: *R. rhipicephali* str. H#J5.

SUPPLEMENTARY TABLE

Slopes of regression line for quantitative polymerase chain reaction analysis

Template[A]	Slope of C_T versus template DNA dilutions
Rickettsia rickettsii str. Taiaçu	- 0,0086
Rickettsia parkeri str. AT#24	- 0,0047
Rickettsia rhipicephali str. HJ#5	- 0,0158

A: template represents DNA samples from Vero cells infected with *R. rickettsii* str. Taiaçu, *R. parkeri* str. AT#24 or *R. rhipicephali* str. HJ#5; C_T: cycle threshold.
